# Posterolateral thoracotomy: unique approach to tricuspid valve replacement after right pneumonectomy

**DOI:** 10.1093/jscr/rjad186

**Published:** 2023-04-18

**Authors:** Austin Hingtgen, Chesney Siems, Stephen J Huddleston, Ranjit John

**Affiliations:** University of Minnesota Medical School, Minneapolis, MN, USA; Division of Cardiothoracic Surgery, Department of Surgery, University of Minnesota, Minneapolis, MN, USA; Division of Cardiothoracic Surgery, Department of Surgery, University of Minnesota, Minneapolis, MN, USA; Division of Cardiothoracic Surgery, Department of Surgery, University of Minnesota, Minneapolis, MN, USA

## Abstract

A 72-year-old patient presented with severe tricuspid regurgitation and patent foramen ovale (PFO) in the setting of severe mediastinal shifting after remote right pneumonectomy. Surgical approach was challenging given the significant herniation of the heart and left lung into the right hemithorax. This report describes tricuspid valve replacement with PFO closure via a right posterolateral thoracotomy and alternative cardiopulmonary bypass cannulation strategy.

## INTRODUCTION

Tricuspid valve replacement (TVR) or repair is the gold-standard treatment for symptomatic, severe tricuspid regurgitation (TR) in patients with right ventricular (RV) dilation or right heart failure and is commonly done with other planned cardiac surgical interventions. Patients with severe TR may present with peripheral edema, ascites or other signs/symptoms of right heart failure. Isolated TVR is uncommon but carries a high risk of early postoperative mortality and morbidity; thus, it is historically done late in the disease course [1]. In general, a median sternotomy or right anterolateral thoracotomy is performed to expose the tricuspid valve. This case report discusses TVR and atrial septal defect (ASD) closure through a redo right posterolateral thoracotomy.

## CASE REPORT

A 72-year-old female presented for surgical management of severe TR and residual patent foramen ovale (PFO) with associated platypnea-orthodeoxia syndrome. She had a right pneumonectomy via right posterolateral thoracotomy 16 years prior for a central carcinoid tumor with an intercostal muscle flap for reinforcement of the right bronchial stump. She subsequently developed platypnea-orthodeoxia syndrome and underwent percutaneous closure of a PFO with an Amplatzer device (Abbott; St. Paul, MN) one year later. However, a left-to-right shunt remained on follow-up echocardiography.

**Figure 1:**
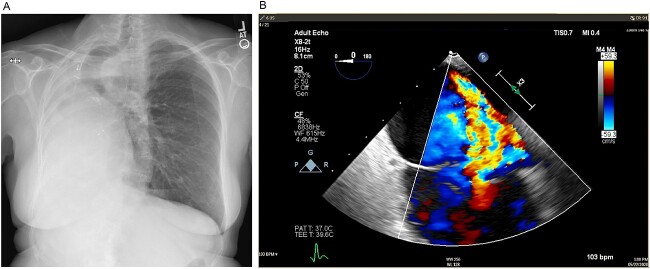
(**A**) Preoperative posteroanterior view chest X-ray showing left to right mediastinal contents shifted and a clear left lung. (**B**) Preoperative transesophageal echocardiogram with doppler showing severe tricuspid regurgitation with no coaptation between the anterior and septal leaflets.

**Figure 2:**
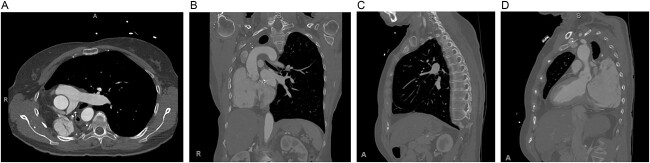
Preoperative computed tomography of the chest. Axial (**A**), coronal (**B**) and sagittal (**C**, **D**) views demonstrating displacement of the heart and left lung into the right hemithorax.

Mild–moderate TR was first noted on transthoracic echocardiogram 5 years after pneumonectomy and continually worsened over the next 10 years until she had wide open severe TR with moderate RV dilation and moderate global reduction in RV function. After multidisciplinary evaluation, TVR was recommended. Preoperative imaging revealed notable mediastinal shift to the right, severe counterclockwise rotation and displacement of the heart to the right and expansion of the left lung into the right hemithorax (Figs 1 and 2).

A right posterolateral thoracotomy was planned for exposure of the right atrium and tricuspid valve, with femoral arterial and venous cannulation, given limited access to the aorta or inferior vena cava (IVC). The patient was placed in a modified left lateral decubitus position with access to the right groin and right posterior chest wall. The femoral vessels were exposed through an oblique femoral incision. A redo posterolateral thoracotomy through the fifth intercostal space was performed. After dividing the latissimus dorsi, the fifth rib was shingled posteriorly and the calcified, previously placed intercostal muscle flap was resected from the chest wall to the posterior mediastinum. After intravenous heparin (300 Units per kilogram) was administered to achieve an activated clotting time over 480 s, femoral arterial and venous cannulation was performed via Seldinger technique and transesophageal echocardiographic guidance. Cardiopulmonary bypass (CPB) was initiated, and an additional superior vena cava (SVC) cannula was placed after it was exposed. An ascending aortic root vent/cardioplegia cannula was also placed. The heart was arrested with cold antegrade blood cardioplegia, and maintenance antegrade cardioplegia was given every 15 min. The patient was cooled to 34°C.

After snaring the cavae, the atrial septum and tricuspid valve were exposed through a right atriotomy from the right atrial appendage extending inferiorly toward the inferior cavo-atrial junction. The previous Amplatz closure of the PFO was densely adherent to the atrial septum and there appeared to be a patent posteroinferior interatrial channel along the fossa ovalis. The tricuspid annulus was dilated without any coaptation of the leaflets, and the RV was severely dilated. The previously placed Amplatzer occluder was removed using sharp and blunt dissection, revealing a large ASD rather than a simple PFO. The ASD was closed with a 2.5 × 3.5 cm ovoid bovine pericardial patch, sewn to the edges of the septal defect with a running 4–0 Prolene suture and confirmed to be occlusive.

**Figure 3:**
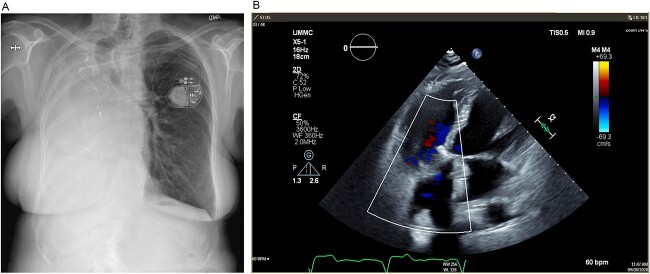
(**A**) Postoperative posteroanterior view chest X-ray. (**B**) Postoperative transesophageal echocardiogram with doppler showing a well-seated bioprosthetic valve in the tricuspid position with a mean gradient of 4 mmHg and no measurable insufficiency.

Turning attention to the tricuspid valve, pledgeted, transannular 2–0 Ethibond sutures were placed circumferentially in everting fashion (atrial side to ventricular side), taking care to place superficial sutures buttressed with the septal leaflet near the atrioventricular node. The annular sutures were brought through the sewing ring of a 33 mm St. Jude Medical Epic bioprosthetic valve that was seated on the annulus, and the annular sutures were secured and cut using the CoreKnot device (LSI Solutions; Minneapolis, MN). Leaflet motion and competence of the valve were confirmed with saline. The right atriotomy was then closed in two layers of 4–0 Prolene. After delivering a dose of terminal warm blood cardioplegia, the aortic cross-clamp was released on low flow CPB. The heart was reperfused, and normal sinus rhythm resumed. With normal biventricular function, the patient was weaned and decannulated of CPB. After achieving hemostasis, the right posterolateral thoracotomy incision was closed. The patient was then taken to the cardiovascular intensive care unit in stable condition. On 8-month follow-up, mean gradient across the tricuspid valve prosthesis was 5 mmHg, RV size and function had returned to normal and the patient had normal exercise tolerance and was feeling well (Fig. 3).

## DISCUSSION

Most open cardiac operations after pneumonectomy are coronary artery bypass grafts with few valve replacements [2]. Prior pneumonectomy poses certain uncommon technical challenges related to exposure for both the valve(s) of interest and cannulation strategy for CPB. This case was challenging given the significant mediastinal shift to the right. Neither median sternotomy nor anterolateral thoracotomy was appropriate due to the displacement of the heart and left lung. The RV abutted the right chest wall with the right atrium in the posterior right pleural space. A posterolateral thoracotomy was necessary to access the tricuspid valve. Additionally, the aorta and IVC were initially inaccessible for cannulation, so the femoral vessels were used, with an additional SVC cannula once it was exposed.

Posterolateral thoracotomy is used frequently in general thoracic operations and for some congenital pediatric cardiac disorders but is rarely utilized in adult cardiac cases [3]. It can be invasive given the division of the latissimus dorsi and serratus anterior muscles. Disadvantages include respiratory complications due the division of these muscles and the associated postoperative pain. Muscle-sparing lateral thoracotomy, where both muscles are mobilized but not divided, can reduce these issues [4]. For clinicians who are presented with similar cases, consider this unique, alternative approach for TVR.

## CONFLICT OF INTEREST STATEMENT

The authors report no conflicts of interest.

## FUNDING

There are no funding sources related to this work.

## INFORMED PATIENT CONSENT

Signed informed patient consent was obtained for approval for publication.

## DATA AVAILABILITY

To the best of our knowledge, all data relevant to this case are found in the manuscript.
